# An Anatomical Approach to Radiofrequency-Assisted Facial Rejuvenation: Beyond the Treatment Gap

**DOI:** 10.1093/asj/sjae232

**Published:** 2025-01-16

**Authors:** Michael J Stein, Neil M Vranis, Sherrell J Aston

## Abstract

**Background:**

Radiofrequency-assisted (RF) facial rejuvenation has become a safe and reliable option for “treatment gap” patients, including (1) patients whose skin laxity is not severe enough to warrant a facelift, yet not mild enough to reliably treat with noninvasive procedures; (2) patients who have already undergone a face or neck lift and have recurrent laxity; and (3) patients who would benefit from a traditional face or neck lift but want to avoid surgery and are willing to accept a more modest improvement without extensive surgical scar burden and recovery.

**Objectives:**

In this study we aimed to educate the reader about providing bipolar RF to various anatomic regions of the face.

**Methods:**

A retrospective review of cases was conducted to assess the safety of zone-specific RF-assisted facial rejuvenation in S.A.'s practice.

**Results:**

RF-microneedling with Morpheus8 was performed on 364 foreheads, 364 periorbita, 353 perioral areas, and 233 jawlines. RF-bipolar with AccuTite was performed on 43 nasolabial folds. There were no cases of hyperpigmentation or hypopigmentation, scarring, or prolonged erythema.

**Conclusions:**

The result of patients treated with the combination of bipolar radiofrequency and fractional bipolar radiofrequency microneedling has expanded RF-assisted facial rejuvenation in our practice. We report on an anatomical approach to RF-assisted facial rejuvenation, as well as expanding indications beyond treatment gap patients.

**Level of Evidence: 4 (Therapeutic):**

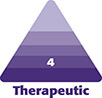

The demand for facial rejuvenation continues to grow and parallels technological advances available for surgeons to improve results with decreased downtime and stigmata of plastic surgery. In 2020, a multicenter, retrospective evaluation of 247 patients undergoing radiofrequency (RF)-assisted facial rejuvenation was published.^1^ This review documented improvements in the appearance of facial and neck aging when combining minimally invasive bipolar RF (FaceTite/AccuTite; InMode Ltd., Lake Forest, CA) and noninvasive fractional bipolar RF (Morpheus8; InMode). In this study we addressed the so-called treatment-gap population in facial rejuvenation, which included 3 patient groups, including (1) young patients whose skin redundancy was not severe enough to justify a traditional excisional procedure (facelift/neck lift), but also not mild enough to treat with liposuction or noninvasive modalities alone; (2) patients who had already undergone a facelift or neck lift who presented with recurrent skin laxity; and (3) patients who would benefit from a facelift or neck lift but wanted to avoid surgery and were willing to accept a more modest improvement. Historically, nonexcisional improvement of facial soft tissue laxity and descent posed a challenge. In this study, however, we demonstrated that improvements in skin appearance and quality were possible with advanced RF-assisted technologies.

The “aging face” is a result of volume deflation and increased dermal and subcutaneous tissue laxity. Energy-based devices address the latter through thermal contraction and subacute neocollagenesis.^1,2^ Bipolar RF energy can be delivered in a transdermal fractional manner through a microneedling device (noninvasive) or as a full-field treatment (minimally invasive). These can be performed independently or concomitantly to obtain the desired results when tailored according to patient anatomy, treatment necessity, and patient desires. A thorough understanding of anatomy, particularly the ligamentous vs fat pad landscape of the face, is critical for optimizing the results of these treatments.

Radiofrequency devices were popularized in nonaesthetic fields such as cardiac catheter ablation and ophthalmology. Now RF-assisted skin tightening of the body, arms, and face continues to grow, with increasing evidence supporting its safety, efficacy, and cost-effectiveness.^1-15^ Volumetric analyses of treated skin support thickening and shortening of the collagen fibroseptal network as well as decreases in the thickness of the subcutaneous fat from heat coagulation.^16^ In facial rejuvenation, when maintaining or even augmenting volume in certain areas to treat the “aging face,” the operator must be precise and intentional with RF thermal energy delivery (ie, power, temperature cutoffs, anatomic location, total energy delivered, and depth of energy delivery). Melting subcutaneous fat of the face may distort contours and lead to unintended downstream sequelae.

The purpose of the present paper is to describe a more focused, anatomical approach to RF-assisted facial rejuvenation, which now expands beyond the traditional treatment gap population outlined in previous reports.^5^ Readers will learn the nuances of RF energy treatment procedures and how to manipulate various treatment parameters according to anatomic locations.

## METHODS

### Radiofrequency Modalities

**Figure 1.**
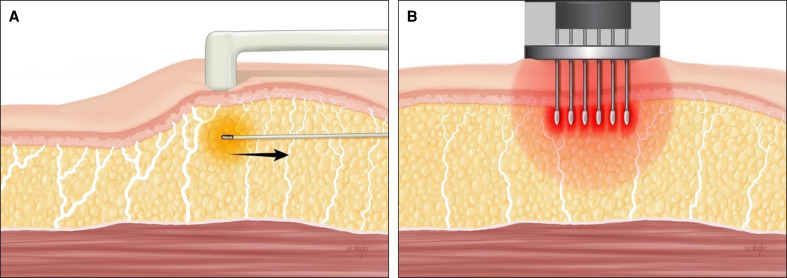
Planes of action of radiofrequency devices. (A) Bipolar radiofrequency probe leading to thickening of the fibroseptal network and coagulation of the subcutaneous fat, resulting in soft tissue contraction. (B) Fractional bipolar microneedling inducing dermal and subdermal cone of coagulation leading to collagen remodeling.

This study was conducted in accordance with the ethical principles stated in the Declaration of Helsinki. Informed consent was obtained from all participants. Consecutive patients undergoing RF-assisted facial rejuvenation in S.A.’s practice were treated with the Embrace protocol (InMode), with a combination of bipolar RF (FaceTite/AccuTite) and fractional bipolar RF (Morpheus8). These 2 RF delivery methods administer energy by different mechanisms and target different depths of the dermal and subdermal tissue (Figure 1A, B). The minimally invasive FaceTite bipolar RF cannula is 1.3 mm in diameter and 13 cm long, and the AccuTite bipolar RF cannula is 0.9 mm in diameter and 6 cm long. For both handpieces the electrical current is directed from the internal tip toward the external, surface probe. The fractional bipolar applicator deploys RF emitting needles with tunable energy levels (1 to 45) at variable programmable depths (in 1-mm increments from 1 to 4 mm). The “number of passes” is another variable, which refers to the number of times the same area undergoes deployment of the bipolar RF microneedles. Fractional bipolar radiofrequency creates controlled zones of dermal injury, narrow at the epidermis, with conical enlargement as the needles descend. It is recommended to reduce the RF power intensity at superficial depths to avoid thermal injury to the epidermis. By doing so, in effect there is a conical distribution of thermal dispersion as greater RF energy is selected at greater needle depths. This is unlike fractional photothermolysis (lasers), which cause thermal injury that tapers at deeper depths.

Initially, rejuvenation of the neck and lower one-third of the face by concomitant bipolar and fractional bipolar RF became the standard of care for treatment-gap patients presenting to our practice.^1^ Now our practice has evolved to a more patient-specific approach that includes a wide array of candidates, beyond traditional treatment-gap categories. Treatment decisions are made to account for the unique anatomical variants between patients, as well as the different topographical anatomy of the face and neck. We therefore routinely modify treatment type and energy delivery based on dermal thickness, fibroseptal network density, subcutaneous fat thickness, and rhytid depth. Gender, age, genetics, and lifestyle contribute to the dermal thickness, amount of subcutaneous fat, and density of the fibroseptal network. RF-assisted treatments must be tailored accordingly.

### Anatomical Approach to Radiofrequency-Assisted Facial Rejuvenation

**Figure 2.**
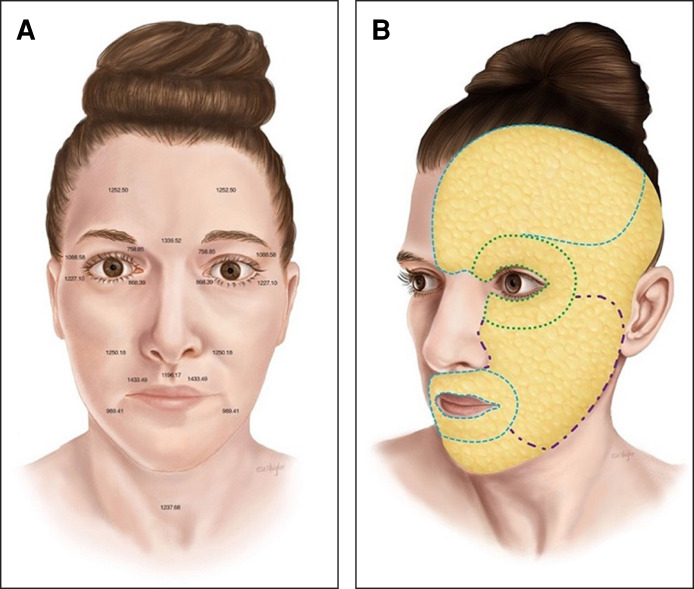
(A) Topographical variations in dermal thickness. The thickness of the skin varies based on the anatomical area of the face. A surgeon's understanding of dermis topographical anatomy is important for adjusting treatment depth with radiofrequency devices. Average dermal thickness (μm) of key treatment zones is presented here (adapted with permission from Chopra et al, ASJ 2015). (B) Topographic variations in subcutaneous fat thickness. When applying FaceTite, surgeons must appreciate the topographical variations in subcutaneous fat thickness of the face. Orienting the bipolar probe safely and with maximal treatment effect requires an understanding of the relative thickness of facial fat across the face (periorbita (green), very thin; forehead and perioral region (blue), thin; malar eminence/cheek (purple), moderate thickness.

**Figure 3.**
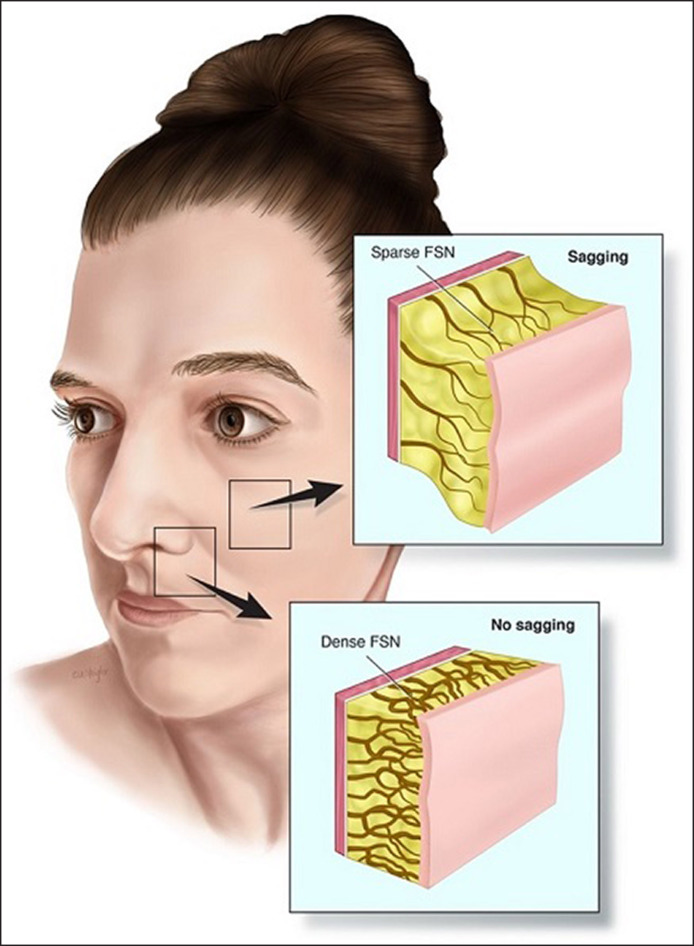
Topographical map of fibroseptal network density. The fibroseptal network (FSN) density varies at different anatomical areas of the face. Malar fat has a thicker subcutaneous layer with longer and less dense FSN arrangement. The FSN anatomy in this anatomical area results in skin sagging over time and deepening of the nasolabial sulcus. The thinner fat layer and dense FSN arrangement of the upper lip contributes to relatively fixed skin which is much less prone to sagging.

An appreciation for facial topographical variations in dermal thickness, fibroseptal network density, fat distribution, and arterial and neural anatomy are critical for surgeons performing facial rejuvenation procedures (Figure 2A, B, Figure 3) of any kinds.^17-29^ Multiple cadaveric and 3-dimensional (3D) volumetric studies have reported regional differences across the face, which are important to the success and safety of applying minimally invasive and noninvasive technologies. Whether performing minimally invasive bipolar RF, fractional bipolar microneedling, or both, the depth, temperature, energy, and number of passes can be modified based on the specific anatomical area being treated to achieve optimal results (Table 1).

**Table 1.** Topographical Approach to Facial Rejuvenation With Radiofrequency Microneedling

Anatomical zone	Energy (J)	Depth	Passes	Treatments	Time between treatments
Forehead	15-20	3 mm	1-2	1-3	4 weeks
		2 mm	2-3		
		1 mm	2-3		
Crows feet	15-20	2 mm	2-3	2-3	4 weeks
		1 mm	2		
Orbital rim, lower eyelids	15-17	2 mm	2	2-3	4 weeks
		1 mm	2		
Cheeks	20-25	3 mm	3	3-4	4 weeks
	15-20	2 mm	3		
	10-17	1 mm	3		
Upper lip, lower lip, chin	17-20	2 mm	3-4	3-4	4 weeks
	12-15	1 mm	3-4		
Fixed perioral resurfacing	15	0.5 mm	1-2	1-3	4 weeks
Neck	15-17	2 mm	3	3-4	4 weeks
		1 mm	3		
Submental	25-30	4 mm	2-3	3-4	4 weeks
	17-20	3 mm	2-3		
	15-17	2 mm	2		
	15-17	1 mm	2		
Nasolabial fold	25-27	4 mm	1-2	2-3	4 weeks
	15-17	3 mm	2-3		
	15-17	2 mm	2-3		
Jowl	20-25	4 mm	2	1-2	4 weeks
	15-17	3 mm	2		
	15-17	2 mm	2		
	15-17	1 mm	2		

## RESULTS

The patient age ranged from 32 to 78 (average age: 58). All patients who participated in this study were female. The patient follow-up time ranged from 3 months to 2 years (average follow-up time: 6 months).

### Forehead

Soft tissue thickness of the forehead is quite variable, but overall quite thin with a very thin layer of subcutaneous fat. Over time, dynamic rhytids develop into static rhytids due to repetitive muscle activation resulting in the aged appearance of the forehead.

Fractional bipolar microneedling is the treatment of choice for the forehead, with the operator modifying the protocol intensity based on the palpated soft tissue thickness. For patients with thin forehead tissue microneedling is performed at 2- and 1-mm depths for 2 to 3 passes, with 50% overlap and an energy of 15J to 20J. When the soft tissue on the forehead is thicker, treatment at 3-, 2- and 1-mm for 2 to 3 passes, 50% overlap, and energy of 15J to 20J is performed.

Subdermal adipose remodeling is anticipated to occur to a greater extent when the forehead is thicker. Even a degree of brow elevation has been observed in some patients. Transverse forehead lines, however, are very difficult to eliminate with RF-assisted treatment alone. Therefore, we routinely use neuromodulators in conjunction to obtain an optimal improvement in patients and find this exceeds the aesthetic of neuromodulators alone. Whereas the neuromodulators address the dynamic rhytids, the RF microneedling treatment improves pore size, dermal thickness, and overall skin quality.^30,31^ High dosages of neuromodulators, or when placed too inferiorly in the frontalis muscle, can cause the eyebrow position to descend. In theory, the dermal contraction from the concomitant RF treatment may also help balance these effects and provide brow stability.

Of 364 foreheads treated with Morpheus8 since 2018, 268 were in the context of isolated full-face RF treatment, and 96 were an adjunct procedure, ancillary to a face and neck lift. There were no minor or major complications with the RF treatments.

### Periorbital Skin

**Figure 4.**
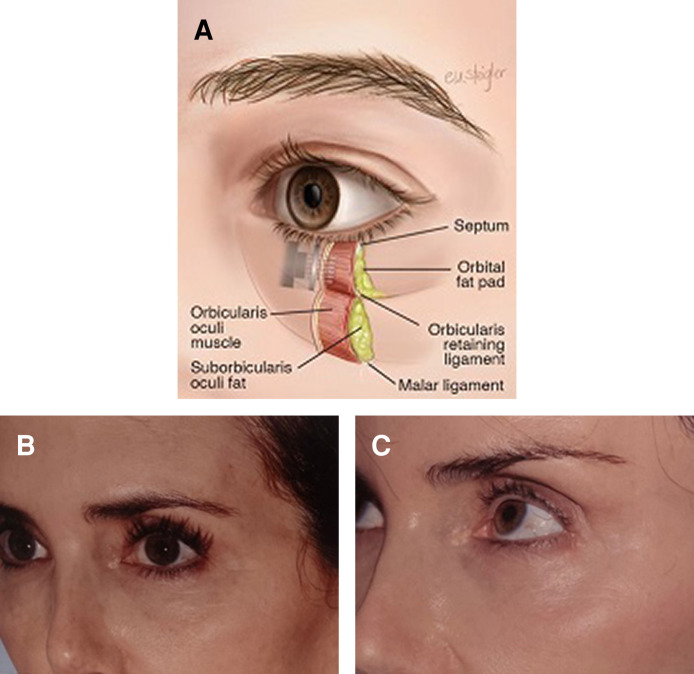
(A) Periorbital anatomy. The infraorbital region has a thin epidermal/dermal layer, scant subcutaneous fat, and a thin orbicularis oculi muscle layer. Safe treatment of this area requires an understanding of the tissue thickness and adjusting the depth of treatment accordingly. (B, C) A 60-year-old female with a total of 4 Morpheus8 periorbital treatments and no concomitant surgical treatment. She was treated according to the protocol in Table 1. The energy was 15J to 20J, with 2 to 3 passes. She had 3 treatments, 4 weeks apart. The first picture (B) was pretreatment. The follow-up picture (C) was at 16 months posttreatment.

Mild to moderate dermatochalasis can be safely and effectively treated with fractional RF microneedling. This area is characterized by very little (if any) subcutaneous fat and the thinnest dermis of the human body.^19^ Our most common treatment protocol includes 3 sessions of fractional bipolar RF microneedling, 4 weeks apart. The Morpheus8 Prime 12-pin tip is always employed because its size lends to precise control of needle placement in this anatomically sensitive area. The inside of the orbital rim is treated with 2 passes at 2 mm and 3 passes at 1 mm. The upper eyelid skin superior to the palpebral sulcus is retracted superiorly such that the skin is on tension and the bony orbit serves as a platform for the treatment. Typically, this area is treated with 2 to 3 passes at 1 mm. For maximal safety, the tip is never directed toward the globe. The lower lid is usually treated at 2 mm and 1 mm with 2 passes at each depth. We will come as close as 2 to 3 mm below the lid margin and lateral canthus. The skin overlying the lateral orbital rim (ie, crow's feet) is then treated with 2 to 3 passes at 2 mm, and 2 to 3 passes at 1 mm. Energy level remains at 20J to 25J throughout. Analogous to the forehead, we have found that concomitant neuromodulators to the lateral orbital orbicularis muscle in addition to the fractionated RF treatment improve skin quality and overall periorbital appearance compared to neuromodulators alone (Figure 4A-C). Intraoperative periorbital treatment is demonstrated in Video 1, in which the patient received stand-alone periorbital treatment under local anesthetic.

Of 364 patients who had periorbital areas treated with Morpheus8 since 2018, 268 were in the context of isolated full-face RF treatment, and 96 received an adjunct procedure, ancillary to a face and neck lift. There were no minor or major complications with the RF treatments.

### Nasolabial Fold

The nasolabial fold is characterized by dense muscular attachments, a condensed superficial musculoaponeurotic system (SMAS) structure, and a denser fibroseptal network inferior to the fold relative to the cheek superior to the fold.^32-35^ The subcutaneous adipose layer above the nasolabial crease is also usually significantly thicker. The aging face is characterized in part by increased laxity of the fibroseptal network leading to malar fat pad descent, thereby deepening the appearance of the sulcus.

**Figure 5.**
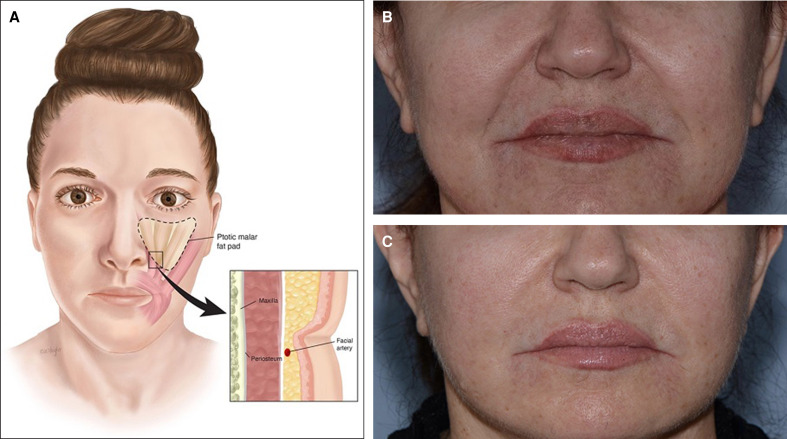
(A) Nasolabial fold anatomy. The nasolabial fold results from dense muscular attachments, a condensed superficial musculoaponeurotic system (SMAS) structure, and a denser fibroseptal network arrangement below compared to above the fold. With aging the thicker malar fat compartments become ptotic, leading to increased depth and definition to the sulcus. (B) A 60-year-old female before and (C) 1 year after the application of AccuTite to nasolabial folds during a facelift. The accompanying video of nasolabial fold treatment (Video 2) is this patient. Although she had a facelift, the nasolabial fold reduction is markedly enhanced by the AccuTite because of the dermolipolysis of the fat in the nasolabial folds. This kind of change is not achieved with a facelift alone.

Facelift procedures redistribute the malar fat pads in a vertical and/or superolateral oblique direction to improve the perceived nasolabial crease. A different technique to address the appearance of a pronounced nasolabial fold involves bipolar RF (AccuTite) through a 1-mm needle puncture at the base of the fold. The operator will observe an immediate improvement due to the thermal contraction of the underlying ligaments. Multiple excursions of the cannula parallel to the nasolabial fold are performed until the desired effect is achieved. Fractional bipolar RF microneedling is then performed: 1 to 2 passes at 4 mm, followed by 2 to 3 passes each at 3-mm and 2-mm depths. This improves skin quality to encourage retraction of the overlying skin of the upper lip-cheek junction and to soften the severity of the nasolabial fold. We follow this approach for severe nasolabial folds (ones that are deep and extend inferior to the lateral lip commissure) as a stand-alone procedure, as well as during a full facelift. When indicated, it is also performed in conjunction with jowl and jawline treatment (Figure 5A, B). An illustration of the nasolabial fold treatment is demonstrated in Video 2.

Since 2020, Accutite was performed for nasolabial folds as a stand-alone procedure in 43 patients and in the context of face and neck lift in 24 patients. There were no minor or major complications.

### Perioral Region

Dermal atrophy and the attenuation of the dense fibroseptal network of the upper lip contribute to pathognomonic changes of the aging lip; lengthening of the prolabium, loss of vermillion height, and static, tangential perioral rhytids. MRI studies illustrate a decrease in the volume and anteroposterior thickness of the upper lip, with histological studies confirming thinning of the dermis, thickening of the subcutaneous layer, and degeneration of the collagen and elastin fibers.^36^ The dermis of the upper lip is one of the thickest areas of the face, and the underlying fibroseptal network forms strong attachment, tethering the dermis to the underlying muscle.^19^ This fixed unit of the face is not improved with a facelift, and fractional bipolar RF treatment is the procedure of choice for patients requesting improvement of the upper lip appearance, but not wanting prolonged downtime as seen with lasers and dermabrasion.

Although it is more common to treat the upper lip in isolation from the lower lip, the entire perioral area requires treatment in a large percentage of patients. As with all aesthetic procedures, adjustments are made based on the quality and apparent thickness of the individual patient's skin and the improvement in appearance needed, allowing for variations according to the patient's pretreatment skin state. Deep, static, vertical lines on the upper lip are particularly displeasing to patients; however, they are challenging to completely efface. We usually perform 4 RF microneedling treatment sessions 1 month apart. The first 2 treatments are at 4-, 3-, 2-, and 1-mm depths with 3 passes at each depth. The third and fourth sessions are at 2 mm and 1 mm, with 4 passes at each depth. When only treating the perioral area, the 12-pin Prime applicator is utilized for precise and even deployment of needles on the convex surface of the upper lip. The fixed resurfacing 0.5-mm-depth tip is also placed for 1 to 3 passes when needed to improve multiple fine lines and wrinkles (Supplemental Figure 1, located online at https://doi.org/10.1093/asj/sjae232).

Of 353 perioral zones treated with Morpheus8 since 2018, 259 were treated as a stand-alone procedure, and 94 cases in the context a face and neck lift. There were no complications seen with these treatments, in contrast to more traditional perioral treatments that include herpetic outbreaks, bacterial infections, and hypopigmentation or hyperpigmentation.

### Jowls/Mandibular Border/Neck

The aging jawline is characterized by attenuation of the fibroseptal support, fat excess, and descent of the 2 mandibular fat compartments posterior to the mandibular ligament.^37,38^ As the fat compartments descend, the sharp mandibular border is obscured, and a sulcus forms around the mandibular ligament, leading to the so-called marionette line anteriorly. Although a surgical release of the ligament and resuspension of fat is an effective way to address the jowl, RF-assisted soft tissue contraction can both reduce the thickness of the subcutaneous jowl fat compartment and strengthen the integrity of the fibroseptal network. Both of these techniques work synergistically to improve mandibular contours.

Both the inferior extent of the descended jowl and the inferior border of the mandible going through the jowl are marked. This designates zone 1, and inferior to the mandibular border represents zone 2. For patients who are having the bipolar RF procedure limited to just the jowl area (and possibly the nasolabial fold) we utilize the AccuTite cannula because it can deliver energy to the entire treatment area. A well-defined lower face-jawline-mandibular border that gently transitions to the adjacent convex and concave contours is the ultimate goal of all facial and neck rejuvenation procedures. This includes the young patient cohort that presents to a plastic surgeon's office desiring an improved cervicomental angle due to genetics, weight fluctuations, or other lifestyle-related anatomy. Precision marking of the entire jowl with the mandibular border dividing zone 1 (above) and zone 2 (below) the mandible in the standing or sitting position is mandatory. We perform application of energy with each stroke upon withdrawal of the cannula in a fanning technique until the desired temperature is reached. Jowl improvement can be seen in real time as the procedure is performed. Subsequent fractional RF microneedling is then performed at 3 mm, 2 mm, and 1 mm directly over the treated jowl area, with 2 passes at each depth. Zone 2, where a mild degree of lipolysis can be beneficial to accentuate contours, can be treated with 3 mm and even 4 mm in some cases.

Most patients benefit from treating the entire mandibular border with the 13-cm 1.9-mm bipolar applicator. Treatment of the entire neck with the bipolar cannula followed by fractional bipolar microneedling can significantly improve neck appearance. Bipolar RF improves the neck skin appearance and cervicomental angle augmenting the jawline. The neck is usually treated bilaterally from the anterior border of the sternocleidomastoid muscle to the midline with 3 access ports; 1 behind each earlobe and 1 in the middle of the submental skin crease. Treatment extends from the mandibular border down to the first cervical skin crease. Temperature settings are set to an internal temperature cutoff of 68 degrees Celsius and external temperature cutoff at 38 to 40 degrees Celsius.

**Figure 6.**
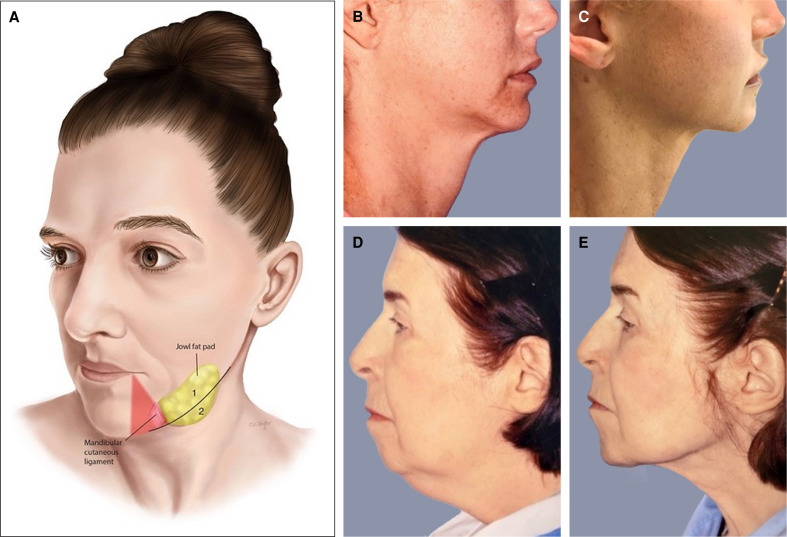
(A) Jowls/mandibular border/neck anatomy. With aging, the jowl fat compartments descend posterior to the mandibular ligament, obstructing a smooth mandibular border. A line marks the mandibular border and the area above and below marked as 1 and 2. A triangle “no go zone” is marked before treatment, representing the relative location of the marginal mandibular nerve. (B, C) A 38-year-old female who had anterior platysmaplasty (submental incision) and 2 sessions of Morpheus8 of the neck, before (B) and after (C). (D, E) This is a 79-year-old patient who had both Morpheus and AccuTite treatment of her neck; (D) pretreatment and (E) posttreatment. Surgically she had a small chin implant. A small submental incision was made and the AccuTite probe was applied to treat the platysma. There was no concomitant surgical platysmaplasty. Muscle became thinner and flatter, and the contour was much improved.

Submental fullness can be very challenging to address when there is any degree of skin laxity, even in younger patients. For each fractional RF microneedling treatment, the settings are as follows: 3 mm, 2 mm, and 1 mm, performing 3 passes at each depth with 50% overlap. A total of 3 to 4 treatment sessions of fractional RF is recommended, 1 month apart, to optimize skin retraction and overall neck contours. Although subdermal adipose remodeling occurs with repeat treatments, it is important to preserve an adequate layer of subdermal fat over the platysma to prevent excessive banding, which is often seen in the aging neck. Therefore, power and needle depth during each treatment session require keen clinical judgement with conservative settings, based on the specific patient's anatomy (Figure 6). Intraoperative videos of the anterior neck treatment and jowl treatment are demonstrated in Video 3 and Video 4, respectively.

Morpheus8 for treatment of the neck was performed in 233 patients since 2018; 77 were in the context a face and neck lift. There were no complications in either the nonsurgical or surgical group. Specifically, there was no evidence of temporary or permanent marginal mandibular nerve palsy with the described protocols.

## DISCUSSION

Radiofrequency-assisted facial rejuvenation has evolved tremendously over the last 4 years of our practice. We have transitioned from a proof-of-concept model to the identification and successful management of “treatment gap” patients, and now to performing bipolar RF and fractional bipolar microneedling RF beyond the treatment gap population.^5^ It has become a key adjunct to a facelift, replacing traditional adjuncts such as lasers, chemical peels, and dermabrasion. Morpheus8 of the upper lip and/or perioral area is fairly standard during facelift surgery. We also apply the Morpheus8 on elevated skin flaps over the neck, mandibular border, and nasolabial folds; however, we refrain from this on elevated skin flaps of the immediate preauricular area, where the skin is thin.

Although surgical excision remains the gold standard for the treatment of moderate to severe facial skin laxity, we have found that the proportion of patients seeking a less invasive procedure with quicker recovery is increasing. A younger demographic is spending more time in the public eye with social media and has a desire for improvement in facial and neck appearance. Many patients present knowing that minimally invasive RF technology can give them an improvement in their specific areas of concern, and they seek physicians with experience in performing these procedures. The terms FaceTite, AccuTite, and Morpheus8 (all products of InMode) are now common in the public vernacular. Due to global marketing efforts, patients will often call the office specifically requesting these procedures, with the short recovery time noted as one of the primary reasons for their interest.

The posttreatment recovery for fractional bipolar radiofrequency is usually minimal. There is no limitation to physical activity immediately after treatment. Mild erythema and edema are expected to last for 1 to 2 days. Patients apply antibiotic ointment 3 times per day for the first 3 days, depending on the extent of the area treated. The microneedle entrance points heal during that time, and patients can resume makeup as they desire. Many patients are able continue work or social engagement throughout the healing process. Ecchymosis is most often due to injection of local anesthesia; when topical anesthesia is utilized there is usually no bruising.

Bruising and edema following bipolar radiofrequency is variable, just as with a full facelift procedure. The overall recovery period is much less than a facelift procedure, because there is no incision to heal, and no stitches to be removed. Posttreatment management of the jowls/mandibular border/neck include our usual facelift compression dressing for 2 days, after which there is no dressing. When concomitant fractional bipolar is performed with a facelift, a small layer of antibiotic ointment is applied just before placing the usual facelift bandage. Whether or not to apply another coat of ointment for an additional day depends on clinical evaluation when the bandage is removed.

In our experience some patients benefit from 2, 3, or 4 sessions of radiofrequency microneedling which are scheduled 4 weeks apart. The subdermal adipose remodeling and collagen/elastin generation continue throughout the entire treatment series, providing cumulative effects. We have observed that although patients see a change in the skin appearance after the first treatment, the continued improvement with each succussive treatment makes them enthusiastic to complete the entire treatment series.

To date we have not observed any burns or scars secondary to bipolar RF. The RF console has built in safety controls of an internal temperature cutoff and external temperature cutoff. The console gives audible and visible cues on the console screen as set temperatures are reached. Treatment continues for 1 minute after target temperatures are reached. The most frequent settings are 68 degrees Celsius internally and 38 degrees Celsius externally.

We have likewise had no scaring or burns with bipolar RF microneedling but are diligent to evaluate the skin appearance with each depth of the treatment and each pass of the handpiece just as one would do with laser or dermabrasion treatments. Energy variations depend on the anatomical zone being treated, as noted in Table 1.

A limitation of this study was the lack of objective, measurable outcomes. The degree of contribution of these adjunct or stand-alone treatments to skin tightening is difficult to quantify. Patient and surgeon safety are 2 important outcomes in aesthetic plastic surgery literature. The main objective here was to report the low complication rate and demonstrate how a very high-volume, experienced surgeon performed these treatments. The goal is that surgeons with less experience with radiofrequency devices can implement these protocols to perform this treatment in a safe, efficient, and reproducible fashion.

## CONCLUSIONS

RF treatment of the various anatomical areas of the face and neck produces soft tissue contraction and improved skin quality appearance. An anatomical approach to facial rejuvenation yields the best results. This requires specific RF type, depth, energy level, and number of treatments to be tailored to the individual patient's anatomy and the specific topographical area of the face being treated.

## Supplemental Material

This article contains supplemental material located online at https://doi.org/10.1093/asj/sjae232.

## Supplementary Material

sjae232_Supplementary_Data

## References

[1] Dayan E, Rovatti P, Aston S, Chia CT, Rohrich R, Theodorou S. Multimodal radiofrequency application for lower face and neck laxity. Plast Reconstr Surg Glob Open. 2020;8:e2862. doi: 10.1097/GOX.0000000000002862 32983756 PMC7489644

[2] Chia CT, Marte JA, Ulvila DD, Theodorou SJ. Second generation radiofrequency body contouring device: safety and efficacy in 300 local anesthesia liposuction cases. Plast Reconstr Surg Glob Open. 2020;8:e3113. doi: 10.1097/GOX.0000000000003113 33133962 PMC7544184

[3] Hurwitz D, Smith D. Treatment of overweight patients by radiofrequency-assisted liposuction (RFAL) for aesthetic reshaping and skin tightening. Aesthetic Plast Surg. 2012;36:62–71. doi: 10.1007/s00266-011-9783-z 21751063

[4] Dayan E, Burns AJ, Rohrich RJ, Theodorou S. The use of radiofrequency in aesthetic surgery. Plast Reconstr Surg Glob Open. 2020;8:e2861. doi: 10.1097/GOX.0000000000002861 32983755 PMC7489578

[5] Theodorou SJ, Del Vecchio D, Chia CT. Soft tissue contraction in body contouring with radiofrequency-assisted liposuction: a treatment gap solution. Aesthet Surg J. 2018;38(Suppl 2):S74–S83. doi: 10.1093/asj/sjy037 29767716

[6] Chia CT, Theodorou SJ, Hoyos AE, Pitman GH. Radiofrequency-assisted liposuction compared with aggressive superficial, subdermal liposuction of the arms: a bilateral quantitative comparison. Plast Reconstr Surg Glob Open. 2015;3:e459. doi: 10.1097/GOX.0000000000000429 26301148 PMC4527633

[7] Theodorou S, Chia C. Radiofrequency-assisted liposuction for arm contouring: technique under local anesthesia. Plast Reconstr Surg Glob Open. 2013;1:e37. doi: 10.1097/GOX.0b013e3182a58c80 25289231 PMC4174202

[8] Duncan DI. Improving outcomes in upper arm liposuction: adding radiofrequency-assisted liposuction to induce skin contraction. Aesthet Surg J. 2012;32:84–95. doi: 10.1177/1090820X11429549 22231416

[9] Stein MJ, Aston SJ. Ancillary procedures to facelift surgery: what has changed? Aesthet Surg J Open Forum. 2023;5:ojad063. doi: 10.1093/asjof/ojad063 38828090 PMC11140481

[10] Gold AH, Pozner J, Weiss R. A fractional bipolar radiofrequency device combined with a bipolar radiofrequency and infrared light treatment for improvement in facial wrinkles and overall skin tone and texture. Aesthet Surg J. 2016;36:1058–1067. doi: 10.1093/asj/sjw086 27474769

[11] Demesh D, Cristel RT, Gandhi ND, Kola E, Dayan SH. The use of radiofrequency-assisted lipolysis with radiofrequency microneedling in premature jowl and neck laxity following facialplasty. J Cosmet Dermatol. 2021;20:93–98. doi: 10.1111/jocd.13824 33128284

[12] Alhaddad M, Wu DC, Bolton J, et al A randomized, split-face, evaluator-blind clinical trial comparing monopolar radiofrequency versus microfocused ultrasound with visualization for lifting and tightening of the face and upper neck. Dermatol Surg. 2019;45:131–139. doi: 10.1097/DSS.0000000000001653 30531187

[13] Angra K, Alhaddad M, Boen M, et al Prospective clinical trial of the latest generation of noninvasive monopolar radiofrequency for the treatment of facial and upper neck skin laxity. Dermatol Surg. 2021;47:762–766. doi: 10.1097/DSS.0000000000003005 33899795

[14] Fabi SG, Niwa Massaki AB, Goldman MP. Clinical efficacy and safety of a monopolar radiofrequency device with comfort pulse technology for the treatment of facial and neck laxity in men. Skinmed. 2016;14:181–185. 27502254

[15] Cook J, DiBernardo BE, Pozner JN. Bipolar radiofrequency as an adjunct to face and body contouring: a 745-patient clinical experience. Aesthet Surg J. 2021;41:685–694. doi: 10.1093/asj/sjaa417 33388742

[16] Zelickson BD, Kist D, Bernstein E, et al Histological and ultrastructural evaluation of the effects of a radiofrequency-based nonablative dermal remodeling device: a pilot study. Arch Dermatol. 2004;140:204–209. doi: 10.1001/archderm.140.2.204 14967794

[17] Lee KW, Kim SH, Gil YC, Hu KS, Kim HJ. Validity and reliability of a structured-light 3D scanner and an ultrasound imaging system for measurements of facial skin thickness. Clin Anat. 2017;30:878–886. doi: 10.1002/ca.22931 28589650

[18] Tsukahara K, Tamatsu Y, Sugawara Y, Shimada K. The relationship between wrinkle depth and dermal thickness in the forehead and lateral canthal region. Arch Dermatol. 2011;147:822–828. doi: 10.1001/archdermatol.2011.158 21768482

[19] Chopra K, Calva D, Sosin M, et al A comprehensive examination of topographic thickness of skin in the human face. Aesthet Surg J. 2015;35:1007–1013. doi: 10.1093/asj/sjv079 26508650

[20] Kim YS, Lee KW, Kim JS, et al Regional thickness of facial skin and superficial fat: application to the minimally invasive procedures. Clin Anat. 2019;32:1008–1018. doi: 10.1002/ca.23331 30629772

[21] Ha RY, Nojima K, Adams WP Jr, Brown SA. Analysis of facial skin thickness: defining the relative thickness index. Plast Reconstr Surg. 2005;115:1769–1773. doi: 10.1097/01.PRS.0000161682.63535.9B 15861089

[22] Nash LG, Phillips MN, Nicholson H, Barnett R, Zhang M. Skin ligaments: regional distribution and variation in morphology. Clin Anat. 2004;17:287–293. doi: 10.1002/ca.10203 15108331

[23] Sakata A, Abe K, Mizukoshi K, Gomi T, Okuda I. Relationship between the retinacula cutis and sagging facial skin. Skin Res Technol. 2018;24:93–98. doi: 10.1111/srt.12395 28868761

[24] Tsukahara K, Tamatsu Y, Sugawara Y, Shimada K. Relationship between the depth of facial wrinkles and the density of the retinacula cutis. Arch Dermatol. 2012;148:39–46. doi: 10.1001/archdermatol.2011.727 22250231

[25] Rohrich RJ, Pessa JE. The fat compartments of the face: anatomy and clinical implications for cosmetic surgery. Plast Reconstr Surg. 2007;119:2219–2227. doi: 10.1097/01.prs.0000265403.66886.54 17519724

[26] Lambros V. Observations on periorbital and midface aging. Plast Reconstr Surg. 2007;120:1367–1376; discussion 1377. doi: 10.1097/01.prs.0000279348.09156.c3 17898614

[27] Rohrich RJ, Pessa JE, Ristow B. The youthful cheek and the deep medial fat compartment. Plast Reconstr Surg. 2008;121:2107–2112. doi: 10.1097/PRS.0b013e31817123c6 18520902

[28] Sieber DA, Scheuer JF 3rd, Villanueva NL, Pezeshk RA, Rohrich RJ. Review of 3-dimensional facial anatomy: injecting fillers and neuromodulators. Plast Reconstr Surg Glob Open. 2016;4:e1166. doi: 10.1097/GOX.0000000000001166 28018775 PMC5172483

[29] Yang HJ, Gil YC, Lee HY. Topographical anatomy of the transverse facial artery. Clin Anat. 2010;23:168–178. doi: 10.1002/ca.20880 19918875

[30] Ren K, Liu H, Li B, Zhou B. Fractional microneedle radiofrequency treatment for enlarged facial pores: a real-world retrospective observational study on 75 patients. J Cosmet Dermatol. 2022;21:6742–6753. doi: 10.1111/jocd.15339 36038248

[31] Sadick NS, Alexiades-Armenakas M, Bitter P Jr., Hruza G, Mulholland RS. Enhanced full-face skin rejuvenation using synchronous intense pulsed optical and conducted bipolar radiofrequency energy (ELOS): introducing selective radiophotothermolysis. J Drugs Dermatol. 2005;4:181–186. 15776775

[32] Snider CC, Amalfi AN, Hutchinson LE, Sommer NZ. New insights into the anatomy of the midface musculature and its implications on the nasolabial fold. Aesthetic Plast Surg. 2017;41:1083–1090. doi: 10.1007/s00266-017-0889-9 28508263

[33] Rubin LR, Mishriki Y, Lee G. Anatomy of the nasolabial fold: the keystone of the smiling mechanism. Plast Reconstr Surg. 1989;83:1–10. doi: 10.1097/00006534-198901000-00001 2909048

[34] Sandulescu T, Spilker L, Rauscher D, Naumova EA, Arnold WH. Morphological analysis and three dimensional reconstruction of the SMAS surrounding the nasolabial fold. Ann Anat. 2018;217:111–117. doi: 10.1016/j.aanat.2018.02.007 29588178

[35] Kwon HJ, O J, Cho TH, Choi YJ, Yang HM. The nasolabial fold: a micro-computed tomography study. Plast Reconstr Surg. 2020;145:71–79. doi: 10.1097/PRS.0000000000006328 31577657

[36] Iblher N, Kloepper J, Penna V, Bartholomae JP, Stark GB. Changes in the aging upper lip–a photomorphometric and MRI-based study (on a quest to find the right rejuvenation approach). J Plast Reconstr Aesthet Surg. 2008;61:1170–1176. doi: 10.1016/j.bjps.2008.06.001 18639513

[37] Reece EM, Rohrich RJ. The aesthetic jaw line: management of the aging jowl. Aesthet Surg J. 2008;28:668–674. doi: 10.1016/j.asj.2008.09.007 19083596

[38] Reece EM, Pessa JE, Rohrich RJ. The mandibular septum: anatomical observations of the jowls in aging-implications for facial rejuvenation. Plast Reconstr Surg. 2008;121:1414–1420. doi: 10.1097/01.prs.0000302462.61624.26 18349664

