# Supplementary figures and images for: An Anatomical Approach to Radiofrequency-Assisted Facial Rejuvenation: Beyond the Treatment Gap
Source: Aesthet Surg J. 2025 Jan 16;45(Suppl 1):S1–9. doi: 10.1093/asj/sjae232 (PMC11862810; doi:10.1093/asj/sjae232)

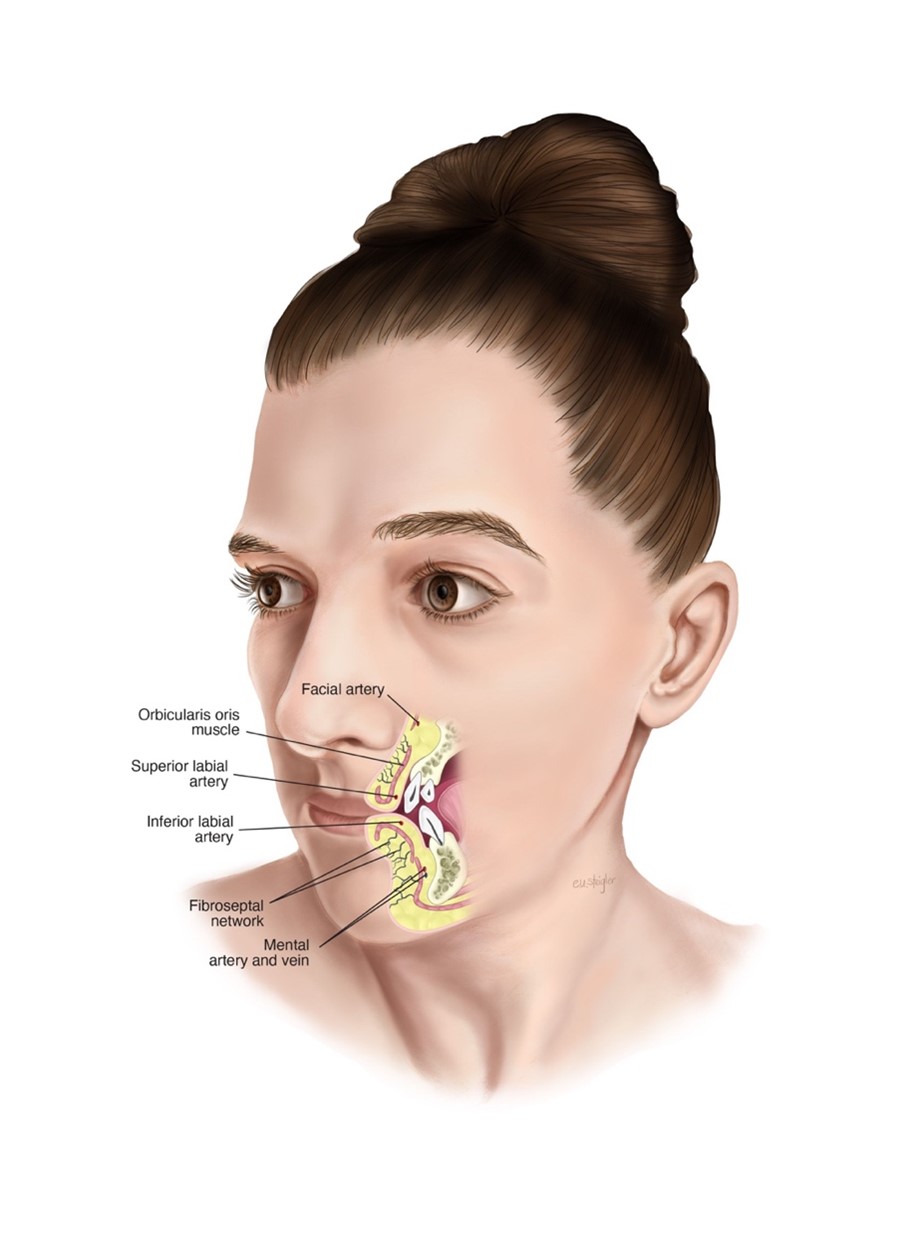

Supplement: sjae232_Supplementary_Data [file sjae232_Supplementary_Data.zip › Fig6a_RF.jpg]

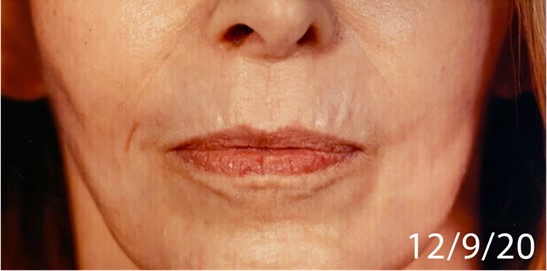

Supplement: sjae232_Supplementary_Data [file sjae232_Supplementary_Data.zip › Fig6b_RF.jpg]

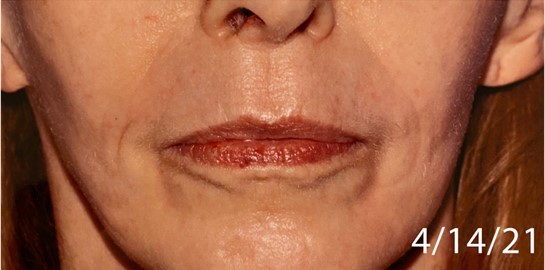

Supplement: sjae232_Supplementary_Data [file sjae232_Supplementary_Data.zip › Fig6c_RF.jpg]
